# An alternative domain-swapped structure of the *Pyrococcus horikoshii* PolII mini-intein

**DOI:** 10.1038/s41598-021-91090-w

**Published:** 2021-06-03

**Authors:** Jennie E. Williams, Mario V. Jaramillo, Zhong Li, Jing Zhao, Chunyu Wang, Hongmin Li, Kenneth V. Mills

**Affiliations:** 1 Department of Chemistry, College of the Holy Cross, 1 College Street, Worcester, MA USA; 2 Division of Genetics, Wadsworth Center, New York State Department of Health, Albany, NY 12208 USA; 3 Department of Pharmacology and Toxicology, College of Pharmacy, University of Arizona, Tucson, AZ 85721 USA; 4 Center for Biotechnology and Interdisciplinary Studies, Rensselaer Polytechnic Institute, Troy, NY USA; 5 Department of Biomedical Sciences, School of Public Health, University at Albany, Albany, NY USA; 6 China Agricultural University, Beijing, China

**Keywords:** Structural biology, Biochemistry, Biochemistry, Structural biology

## Abstract

Protein splicing is a post-translational process by which an intein catalyzes its own excision from flanking polypeptides, or exteins, concomitant with extein ligation. Many inteins have nested homing endonuclease domains that facilitate their propagation into intein-less alleles, whereas other inteins lack the homing endonuclease (HEN) and are called mini-inteins. The mini-intein that interrupts the DNA PolII of *Pyrococcus horikoshii* has a linker region in place of the HEN domain that is shorter than the linker in a closely related intein from *Pyrococcus abyssi*. The *P. horikoshii* PolII intein requires a higher temperature for catalytic activity and is more stable to digestion by the thermostable protease thermolysin, suggesting that it is more rigid than the *P. abyssi* intein. We solved a crystal structure of the intein precursor that revealed a domain-swapped dimer. Inteins found as domain swapped dimers have been shown to promote intein-mediated protein alternative splicing, but the solved *P. horikoshii* PolII intein structure has an active site unlikely to be catalytically competent.

## Introduction

**Figure 1:**
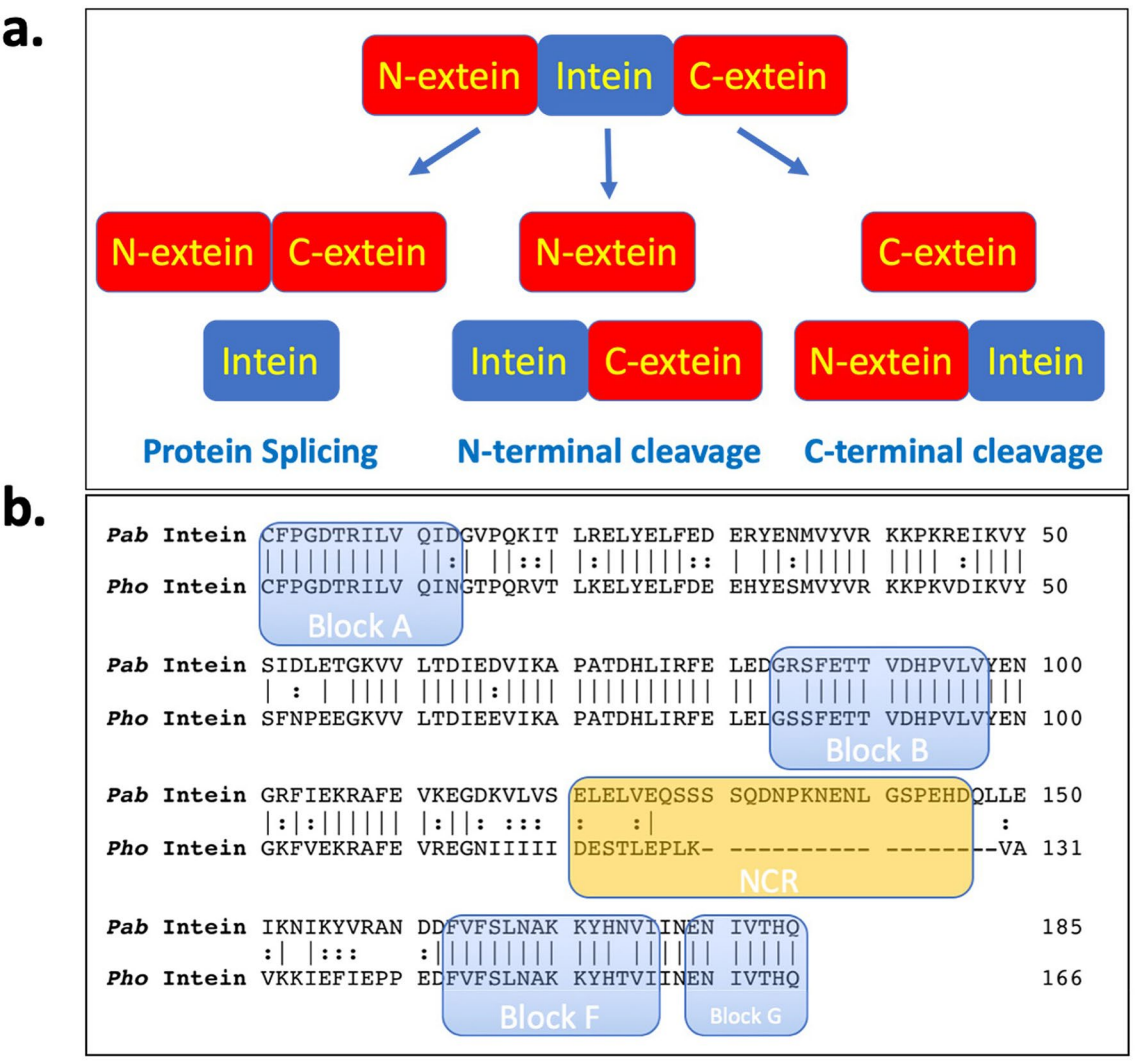
(**a**) The process of protein splicing and splicing side-reactions and (**b**) Comparison of intein sequences. (**a**) Protein splicing is facilitated by an intein, an intervening protein that interrupts the N-extein and C-extein. Splicing results in the ligation of the N-extein and C-extein and excision of the intein. Thiolysis or hydrolysis of the thioester formed in either steps 1 or 2 of splicing can result in N-terminal cleavage if the third step of splicing is blocked by mutation. Premature cyclization of the C-terminal Gln or Asn of the intein (step 3 of splicing), uncoupled from steps 1 or 2, can result in C-terminal cleavage. (**b**) A sequence comparison of the DNA PolII inteins from *Pyrococcus abyssi* (*Pab*) and *Pyrococcus horikoshii* (*Pho*). The EMBOSS water tool was used to align the inteins. Identical residues are marked with a pipe and similar residues are marked with a colon. Intein blocks A and B make up the N-terminal segment of the active site, and blocks F and G make up the C-terminal segment of the active site. Blocks C, D, and E are missing as both inteins are so-called “mini-inteins” that have an NCR in place of a homing endonuclease domain.

Inteins catalyze their own excision from flanking polypeptides, or exteins, via the process of protein splicing (Fig. 1a)^1^. Protein splicing is a four-step process. First, the peptide bond linking the N-extein and intein is converted to a thioester by nucleophilic attack of the N-terminal Cys of the intein on the adjacent peptide bond. Second, the N-terminal Cys of the C-extein facilitates a *trans*-thioesterification reaction by which the N-extein is transferred from the side chain of the first residue of the intein to the side chain of the first residue of the C-extein. Cleavage of either of these thioester intermediates by exogenous thiols or water results in separation of the N-extein from the intein-C-extein fusion protein in a process called N-terminal cleavage. (In other inteins these residues may be Ser, resulting in oxygen ester intermediates.) Third, the C-terminal Gln or Asn of the intein is cyclized, resulting in cleavage of the peptide bond linking the intein and exteins and release of the intein with a C-terminal aminoglutarimide or aminosuccinimide, and release of the exteins linked by a thioester. Premature Gln or Asn cyclization can lead to C-terminal cleavage uncoupled from splicing. Finally, the thioester linking the exteins is spontaneously converted to the more favorable amide, and the intein’s C-terminal residue may be hydrolyzed to Gln/Asn or *iso*-Gln/*iso*-Asn.

Many inteins are interrupted by nested homing endonuclease (HEN) domains, which separate the inteins into N-terminal segments (with conserved sequence motifs A and B) and C-terminal segments (with conserved sequence motifs F and G). (See Fig. 1b.) LAGLIDADG-family homing endonuclease domains, similar to those found in mobile introns, comprise the conserved intein motifs C, D, E, and H. Other inteins naturally lack HEN domains and are called mini-inteins^2,3^, including the inteins that interrupt the *Pyrococcus abyssi* and *Pyrococcus horikoshii* DNA Polymerase II (i.e., the *Pab* PolII and *Pho* PolII inteins)^4,5^. In some cases, mini-inteins have been engineered from HEN-containing inteins by rational design and/or directed evolution. The sequence that replaces the HEN-domain, or is present in the absence of a native HEN-domain, has been termed the non-conserved region, or NCR^6^. In some cases, a native HEN domain can be replaced with an NCR without significantly altering the efficiency of splicing^2,7–9^, but in other cases such replacement can be deleterious^10,11^.

We previously have shown that the *Pab* PolII intein can be isolated as an unspliced precursor and induced to splice in vitro at elevated temperatures and in the presence of reducing agent^4,12^. Other inteins from thermophiles previously had been shown to promote temperature-dependent in vitro splicing^13,14^. We have reported the NMR solution structures of an excised *Pab* PolII intein^15^. Most regions of the *Pab* PolII intein had very high order parameters, as determined by ^15^N spin relaxation, which suggests that the intein has a very rigid backbone at 40 °C. However, the NCR for this intein is highly disordered, as determined by the order parameters. The 26-residue NCR also displays a very high RMSD between the 20 conformers in the NMR structure ensemble and the mean coordinates.

We attempted to find conditions to crystallize the unspliced *P. abyssi* intein and were not successful. We hypothesize that this was due in part to the disorder in the NCR. We observed that the *Pho* PolII intein has a shorter NCR^5^, so we predicted that the NCR might be less flexible and thus make the intein a better target for crystallization. Given the sequence similarity to the *Pab* PolII intein, we predicted that it also would promote temperature-dependent splicing, despite a report from a screening assay that it was inactive^16^.

In this report, we show evidence that the shorter NCR may indeed result in a more rigid intein structure, as the intein requires higher temperatures to facilitate splicing and intein side reactions and is less sensitive to cleavage by thermolysin. We were able to discover crystallization conditions, but the solved structure reveals a domain-swapped dimer with the active site in a conformation that is unlikely to be catalytically competent.

## Results and discussion

We hypothesized that the *Pho* PolII intein would react similarly to the *Pab* PolII intein given their sequence similarity (Fig. 1b). Using the EMBOSS water tool^17^, the inteins are 64% identical and 78% similar by amino acid sequence. Excluding the gap at the NCR, the inteins are 72% identical and 87% similar. Blocks A and B define the highly conserved intein regions at the intein N-terminus and blocks F and G at the C-terminus,these blocks fold to constitute the intein active site^18^. The two inteins differ by only one residue in blocks A, B, and F, and are identical in block G. This suggests that the active site of each intein is very similar, and that differences in temperature-dependent activity between the inteins may be due to the stability of the protein fold.

**Figure 2:**
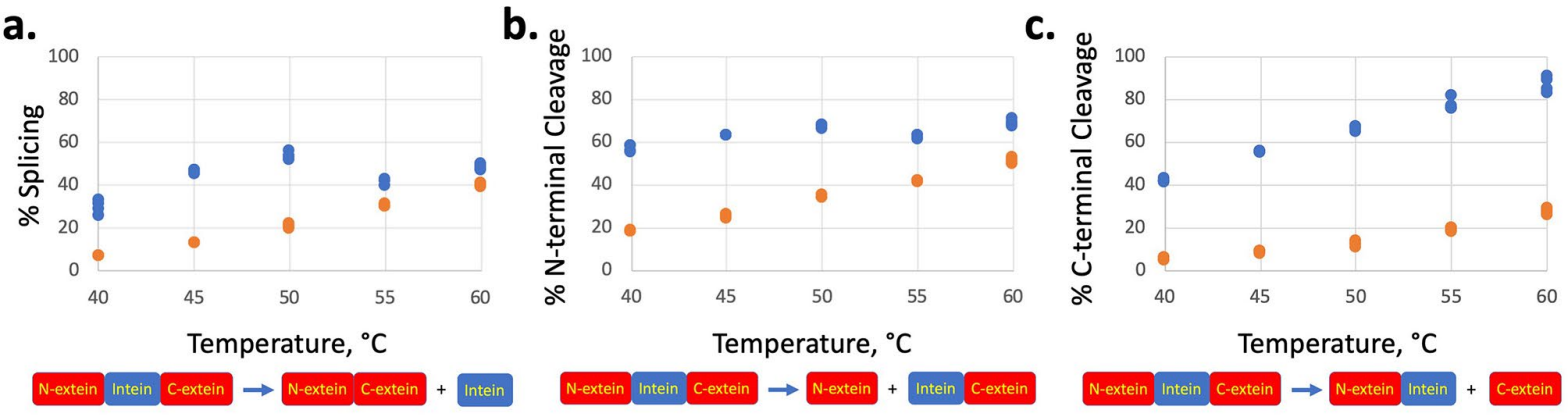
Protein splicing and splicing side reactions of the *P. abyssi* and *P. horikoshii* PolII inteins. Activity of the *Pab* PolII intein in blue and the *Pho* PolII intein in red. Each point represents analysis of a single experiment, and four experiments were used at each temperature point from one purified precursor protein. The extent of each reaction was measured by SDS-PAGE stained with Coomassie blue, with analysis of percentage of activity by densitometry using ImageJ. (**a**) Splicing as a function of temperature was measured by SDS-PAGE. Precursor intein fusion protein MIH-QN, which has a C-terminal Gln to Asn mutation to promote faster splicing, was incubated in buffer A supplemented with 2 mM TCEP and 5 mM EDTA for 8 h at the indicated temperatures. (**b**) N-terminal cleavage as a function of temperature measured by SDS-PAGE. Precursor intein fusion protein MIH-QACA, which has Cys + 1 to Ala and C-terminal Gln to Asn mutations to block the second and third steps of splicing, was incubated in buffer A supplemented with 100 mM DTT and 5 mM EDTA for 2 h at the indicated temperatures. (**c**) C-terminal cleavage as a function of temperature as measured by SDS-PAGE. Precursor intein fusion protein NIH-C1AQN, which has a Cys1 to Ala mutation to prevent the first step of splicing and a C-terminal Gln to Asn mutation to promote faster C-terminal cleavage, was incubated in buffer A supplemented with 2 mM TCEP and 5 mM EDTA for 5 h at the indicated temperatures. See Supplementary Figs. S1–S3 for SDS-PAGE experiments.

To examine the influence of temperature on the extent of intein activity in vitro, we purified unspliced intein fusion proteins of both inteins. The fusion proteins consist of an N-terminal *E. coli* Maltose Binding Protein (MBP), the seven C-terminal residues of the N-extein, the respective inteins, and the C-terminal sequence C-D-G-D-E-D-H_6_, representing the first six native C-extein residues and His-tag. The *Pho* PolII intein has 166 residues and the *Pab* PolII intein has 185 residues, and they share the same immediate flanking extein sequences. To study N-terminal cleavage of the thioester formed in step one of the splicing mechanism in isolation, we made mutations to Ala of the C-terminal Gln of the intein and N-terminal Cys of the C-extein. To study C-terminal cleavage via cyclization of the C-terminal Asn in isolation, we made a fusion protein with the N-extein replaced with the sequence M-H-A-A-K-R-N, along with mutations of Cys1Ala and the final Gln to Asn in the intein. We have previously shown that in vitro splicing of the *P. abyssi* PolII intein is temperature dependent, and requires reduction of a disulfide bond linking Cys1 and Cys + 1^4,12,19^. N- and C-terminal cleavage of the intein also requires incubation at high temperature. The rate and extent of splicing and isolated C-terminal cleavage are greater with substitution of the highly conserved C-terminal Asn for the native C-terminal Gln^12^. Here, we show that splicing and the uncoupled cleavage reactions of the *Pho* PolII intein are also temperature dependent, and are generally less active than the corresponding *Pab* PolII inteins at lower temperatures (Figs. 2, S1–S3 for SDS-PAGE experiments). This supports our hypothesis that the structure of the *Pho* PolII intein might be more rigid than the *Pab* PolII intein and require a higher temperature for activity, given the reduced length of the NCR that is highly flexible in the *P. abyssi* PolII NMR structural ensemble^15^.

**Figure 3:**
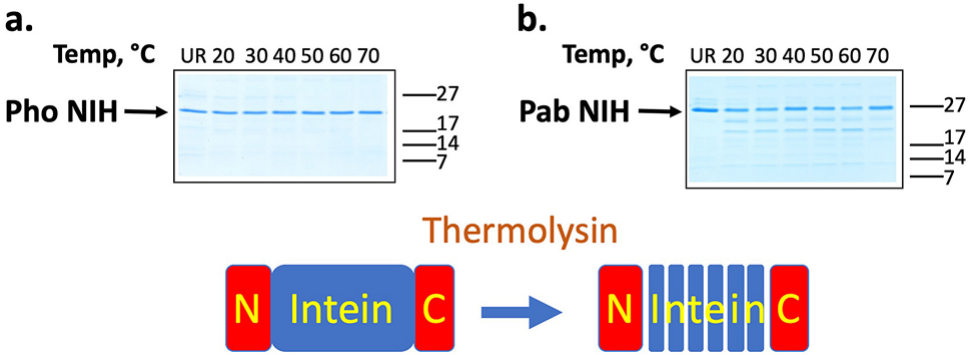
Analysis of intein stability of the *Pho* PolII and *Pab* PolII inteins by temperature-dependent thermolysin digestion. Precursor intein fusion protein NIH-C1A-QACA of (**a**) *Pho* PolII or (**b**) *Pab* PolII was incubated in buffer C first for 10 min and then for 1 h after the addition of 30 ng/mL thermolysin at the indicated temperatures, and analyzed by SDS-PAGE stained with Coomassie blue. Lane UR is unreacted precursor fusion protein. NIH-C1A-QACA has mutations of Cys1, the C-terminal Gln, and Cys + 1 to Ala to prevent splicing reactions. See Supplementary Fig. S4 for full SDS-PAGE experiments.

We also compared the two inteins by their relative sensitivity to degradation by the thermostable protease thermolysin, which digests proteins in unstructured regions^20^. Thermolysin has been used to measure the stability of minimized versions of the *Mycobacterium tuberculosis* RecA intein toward thermal unfolding^21^, and we recently used thermolysin analysis to determine the contribution of mutations in the extremophile hairpin (EXH) motif comprised by β strands 4 and 5 to the stability of the fold of the *Pab* PolII intein^22^. Incubation with thermolysin leaves the *Pho* PolII intein uncleaved under conditions that lead to controlled digestion of the *Pab* PolII intein (Figs. 3, S4), suggesting that the flexible regions degraded in the *Pab* PolII intein are not similarly flexible in the *Pho* PolII intein.

**Figure 4:**
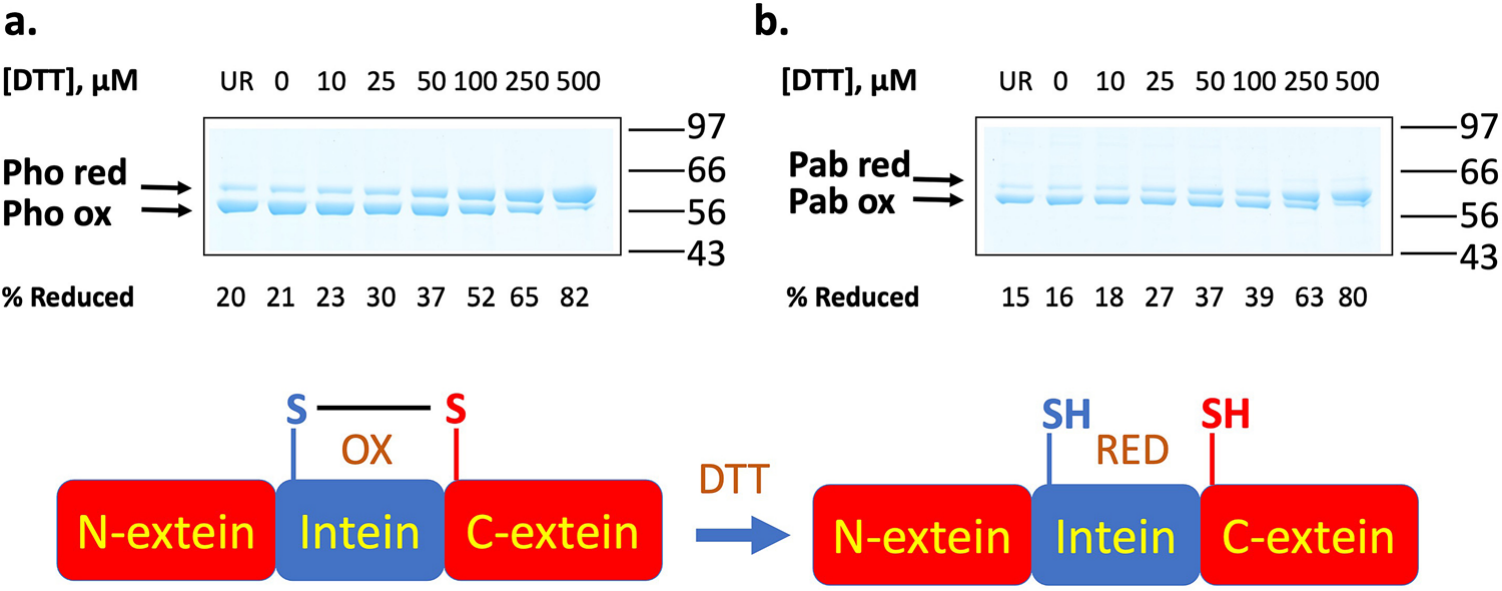
Analysis of disulfide bond strength of the bond between Cys1 and Cys + 1 of the *Pho* PolII and *Pab* PolII inteins. Precursor intein fusion protein MIH of the (**a**) *Pho* PolII or (**b**) *Pab* PolII intein was incubated with the indicated concentrations of DTT for 30 min at 20 °C, and analyzed by SDS-PAGE without DTT in the loading buffer and stained with Coomassie blue. The percentage of reduced protein was analyzed by densitometry using ImageJ. See Supplementary Fig. S5 for the full SDS-PAGE experiments.

The *Pab* PolII intein has a disulfide bond that links the catalytical Cys residues in the active site, Cys1 and Cys + 1^19^. This indicates that these residues, 186 residues apart in the primary sequence, are immediately adjacent in the active splicing precursor. This explains the requirement for reducing agent during in vitro splicing. The disulfide bond alters the migration of the unspliced protein via SDS-PAGE under non-reducing conditions,this aberrant migration can be resolved by the addition of DTT to the SDS-PAGE experiment. In Fig. 4, we show that the *Pho* PolII intein also displays aberrant migration under non-reducing conditions, suggesting that there is also a disulfide bond linking Cys1 and Cys + 1, the only two Cys residues in the protein. (See also Fig. S5.) Given the relative similarity of the effect of DTT on reduction of the disulfide bonds, it appears that the bonds are of similar strengths, suggesting that the native active site of the *Pho* PolII intein also brings the catalytic Cys residues in close contact.

Given our hypothesis that the *Pho* PolII intein may be more rigid, we hoped that we would find conditions that permitted solution of the structure of an unspliced precursor by x-ray crystallography. Finding such conditions had eluded us for the *P. abyssi* PolII intein, perhaps due to the flexible NCR. We were pleased to discover that we could reproducibly isolate crystals of an unspliced *Pho* PolII intein fusion protein with an N-extein of M-H-A-A-K-R-N fused to the 166-residue intein and the C-extein C-D-G-D-E-D-His_6_. However, we were surprised that we were unable to infer the phasing via molecular replacement, as we anticipated that the *Pho* PolII intein structure would be similar to the canonical intein fold^23,24^. Instead, we resorted to single anomalous diffraction with selenomethionine-derivatized *Pho* PolII intein.

**Figure 5:**
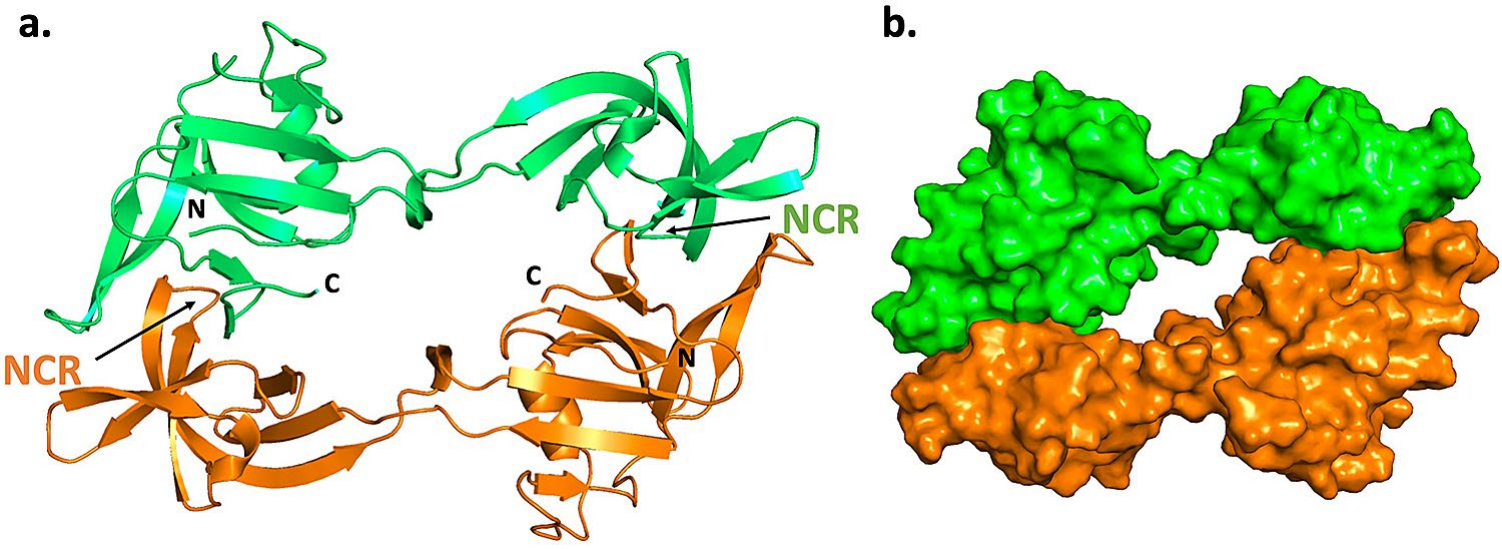
Crystal structure of the *Pho* PolII intein as a domain swapped dimer. (**a**) Ribbon diagram of the two monomers of the *Pho* PolII intein with the N- and C-termini indicated (PDB code 5BKH). The data begin at N-extein residue Arg-3 to C-extein residue Cys + 1. The non-conserved region (NCR) is shown on each monomer. (**b**) Van der Waals representation of the *Pho* PolII intein precursor, colored by chain, to demonstrate the dimer interface. Figures were made with PyMOL 2.4.0.

Surprisingly, solution of the crystallography data revealed that the *Pho* PolII intein precursor was present in the crystal as a domain swapped dimer. The canonical HINT fold has pseudo two-fold symmetry^24^. The intein structure we observed consisted of monomers opened between the two symmetry-related units, with the N- and C-termini of the inteins on the same lobe of the monomer (Fig. 5a). We could visualize three N-extein residues in the electron density map. Residues Arg-3 to Pro71 and Phe144 to Cys + 1 are in one lobe of the monomer and residues Leu 76 to Ile138 are on the other lobe. Analysis of the van der Waal contact area by PyMol suggests a substantive dimer interface area of about 1560 Å^2^ (Fig. 5b).

**Figure 6:**
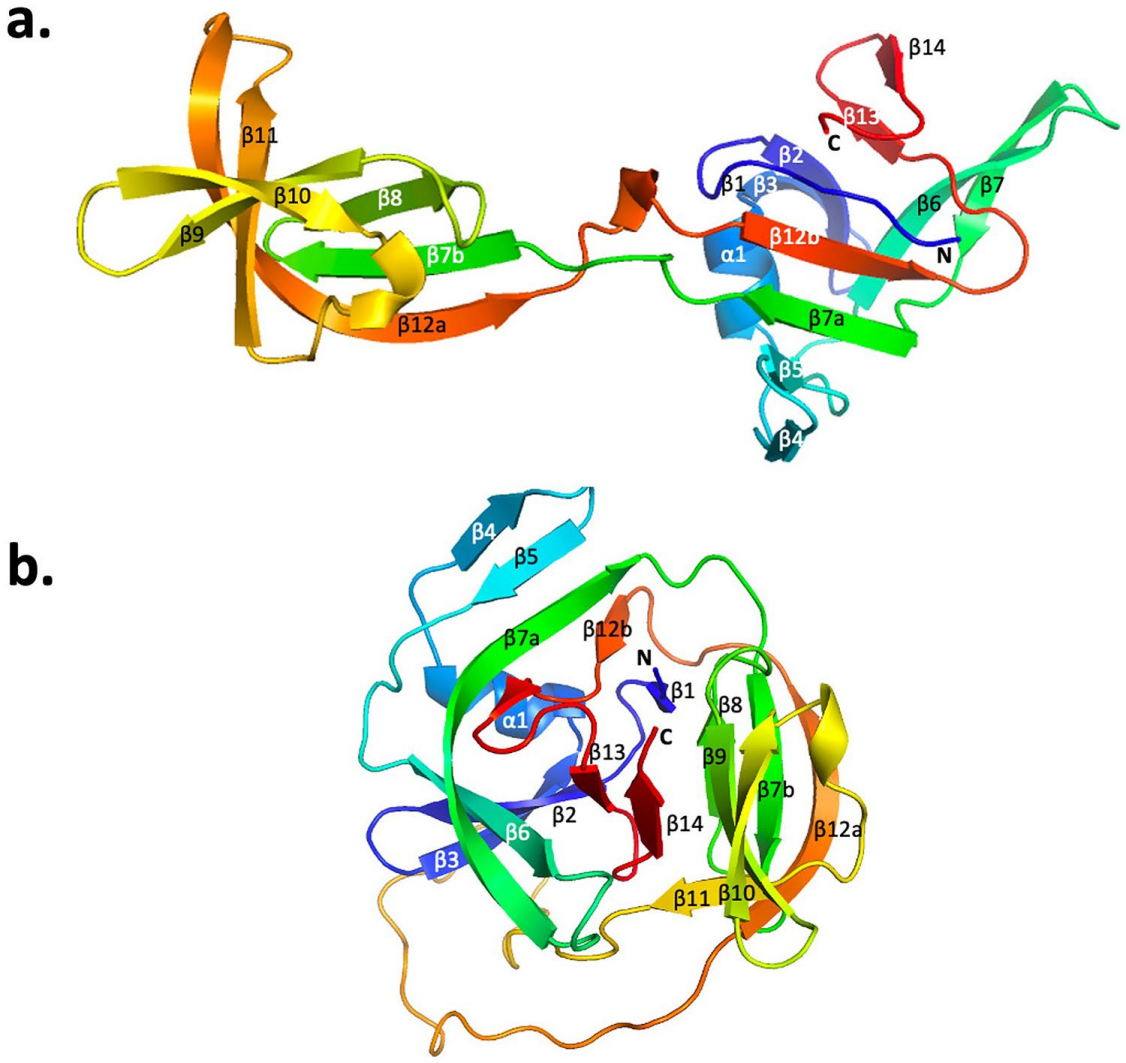
Crystal structure of a single monomer of the *Pho* PolII intein and the *Pab* PolII intein crystal structure. (**a**) Single monomer of the *Pho* PolII intein (PDB code 5BKH). (**b**) Structure of the solution closest to the mean of the NMR ensemble of the *Pab* PolII intein (PDB 2LCJ). Figures were made with PyMOL 2.4.0. The rainbow coloring progresses from N terminus (blue) to C terminus (red).

In Fig. 6, we compare the structure of one monomer of the *Pho* PolII intein to the closest structure to the mean among the NMR structure ensemble of the *Pab* PolII intein^15^. Sequentially, we can observe for the *Pho* PolII intein that the last three residues of the N-extein are followed by a short β-strand from residues intein Phe2 to Pro3 (using the first Cys residue of the intein as residue 1 in the numbering scheme). This is followed by strands β2 and β3 from Arg7 to Ile12 and Thr15 to Thr20, respectively, and then an α-helix from Leu21 to Leu27, with one side packed against the intein β-sheet (Leu21, Leu24, Tyr25) and the other side facing solvent (Lys22, Glu23, and Glu26). Next, strands β4 (Phe28 to Glu34) and β5 (Val37 to Pro44) make up the extremophile hairpin motif^22^. This is followed by a twisted hairpin of β6 (Ile47 to Asn 53) and the first half of β7 (β7a, Lys58 to Ala 70). The conserved intein β7 is contiguous in some intein structures, but is also split in the *Pab* PolII intein structure^15^. Strand β7a is disrupted at Pro71, which leads into a passage into the other lobe of the monomer. Strands β7b (Leu76 to Leu81) and β8 (Ser86 to Thr89) make up a β-hairpin and a β-sheet with β12a. Next, stands β9 (Pro94 to Glu99) and β10 (Lys102 to Arg107) comprise another twisted β-hairpin, and lead into a short 3_10_ helix (Ala108 to Glu110). This leads into another twisted beta-hairpin between stand β11 (Asn115 to Ile120) and β12a (Pro127 to Ile138). The NCR in the *Pab* PolII intein is located between these two β-strands. Strand β12 is interrupted, as it is in the *Pab* PolII structure, but in this case the interruption leads to the passage back into the other lobe of the monomer, including a short 3_10_ helix from residues Pro141 to Asp143. Strand β12b (Val145 to Ala 150) is inserted into the β-sheet in the first lobe anti-parallel to β7a. Finally, β13 (Val156 to Ile 158) and β14 (Asn161 to Val 163) make a β-hairpin.

**Figure 7:**
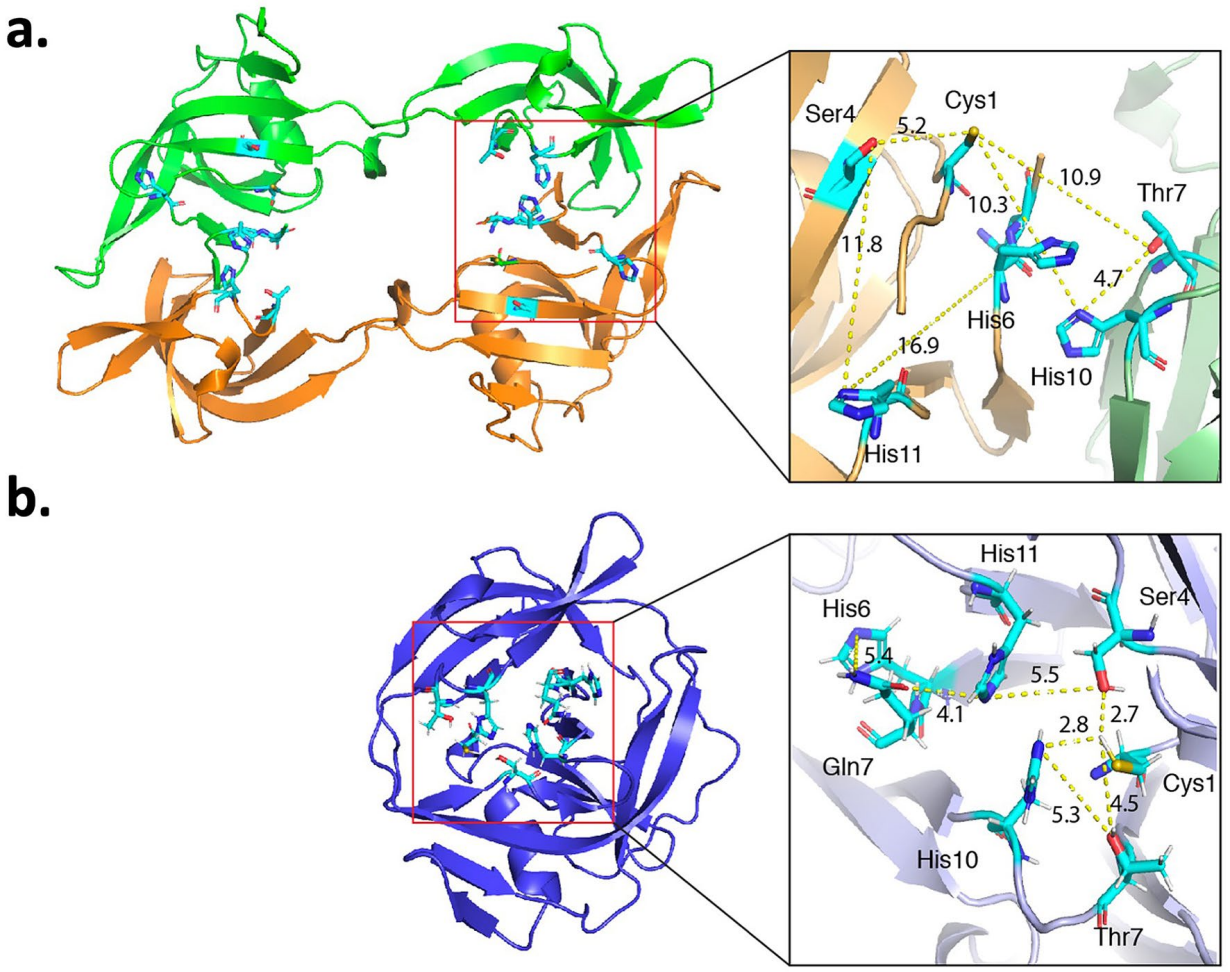
Active site organization of the *Pho* PolII and *Pab* PolII inteins. Distance between key residues in the active site of (**a**) the *Pho* PolII intein domain swapped dimer (PDB code 5BKH) and (**b**) the *Pab* PolII intein NMR structure for the solution closest to the mean of the NMR ensemble (PDB 2LCJ). Cys1 is in intein block A and is the nucleophile in step 1 of splicing. Thr7 and His10 of intein block B help to catalyze step 1 of splicing. Gln7 of block G is the ultimate intein residue that is cyclized in step three of splicing; this step is catalyzed by the penultimate block G His and the conserved His11 of block F. Residue Ser4 of block F helps to coordinate the steps of splicing. Figure was made with PyMOL 2.4.0.

The active site of the domain swapped dimer is compromised, making it unlikely that this conformation of the intein is catalytically competent (Fig. 7). In Fig. 7, the *Pab* PolII and *Pho* PolII intein structures are shown in the same orientation, as demonstrated by the inset from the structures to the left. It is therefore noteworthy that the conformations of the active sites are considerably different. The thiols of Cys1 and Cys + 1 are 3.4 Å apart in the *Pho* PolII structure, suggesting that there could be a disulfide bond present in each monomer, as we observe experimentally (Fig. 4) and in the *Pab* PolII monomer. The peptide bond linking Asn-1 and Cys1 is in the fully *trans* configuration with a dihedral angle of 177.1°, indicating no strain in this scissile bond for the *Pho* PolII intein, which has been suggested as a potential means for altering the equilibrium position between the amide and thioester in step 1 of splicing^25–27^. Other key aspects of the active site are significantly disrupted in comparison to the active site of the *Pab* PolII intein (PDB code 2LCJ, Fig. 7b). The conserved block B intein residues His93 (position B10) and Thr90 (position B7) play an important role in promoting the first step of splicing of the *Pab* PolII intein^15^. In the structure of the excised *Pab* PolII intein, the εN of His93 is 2.8 Å from the thiol of Cys1 and the side chain hydroxyl of Thr90 is 4.5 Å away. In the *Pho* PolII structure, these distances are 10.3 Å and 10.9 Å, respectively. Another key residue in the splicing of the *Pab* PolII intein is Ser166, which is in the key 4^th^ position of block F and can serve to coordinate the steps of splicing. The side chain of this residue is 2.7 Å from Cys1 and 5.5 Å from the side chain of the block F His. In the *Pho* PolII structure, these distances are 5.2 Å and 11.8 Å, respectively. The block F His can help to promote the third step of splicing, perhaps by deprotonating the side chain amide of the C-terminal Gln or Asn. Despite the *Pab* PolII structure being of an excised intein, the block F His is only 4.1 Å from the side chain of the C-terminal Gln. In the *Pho* PolII structure, this distance is almost 17 Å.

**Figure 8:**
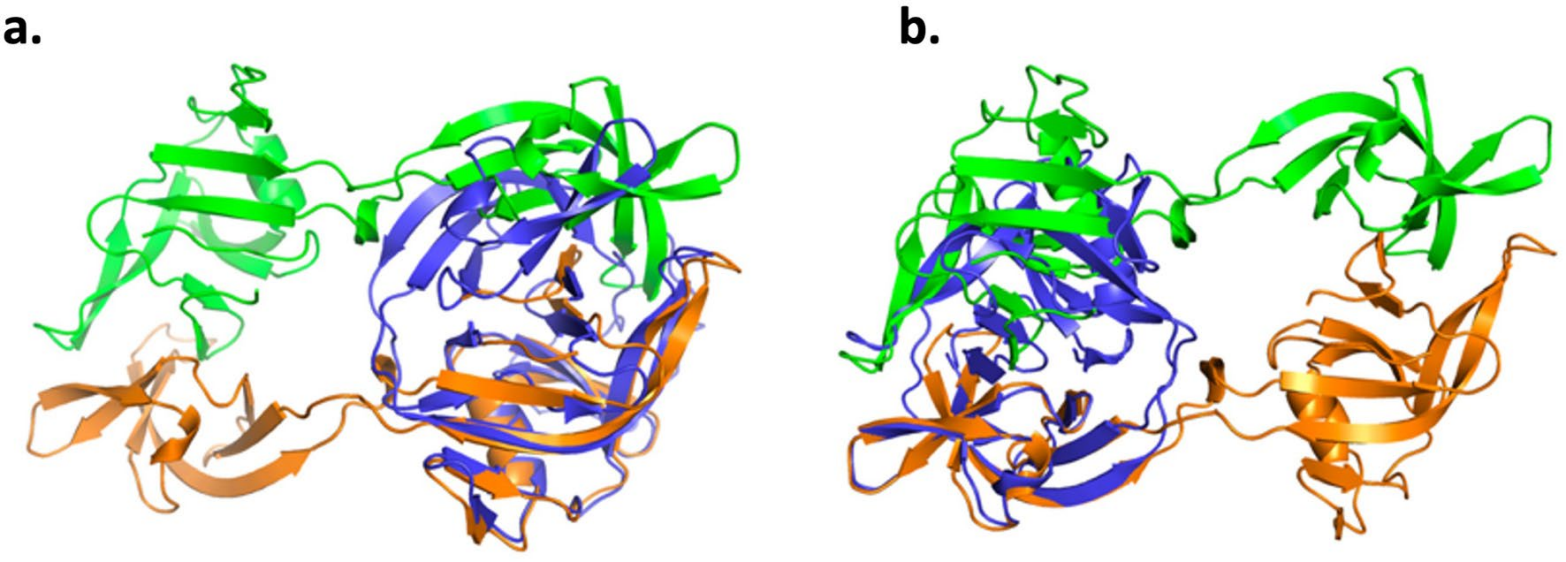
Structural alignment of the *Pho* PolII intein and the *Pab* PolII intein. Structural alignment of the *Pab* PolII intein (blue, PDB code 2LCJ) and one monomer of the *Pho* PolII intein (orange, PDB code 5BKH). (**a**) Structural alignment using the align function in PyMOL to align residues Arg6 to Thr81 of the *Pho* PolII intein orange monomer with the *Pab* PolII intein, resulting in a RMSD value of 0.93 Å. (**b**) Structural alignment using the align function in PyMOL to align residues Phe87 to Asp151 of the *Pho* PolII intein orange monomer with the *Pab* PolII intein, resulting in a RMSD value of 1.21 Å. Figure was made with PyMOL 2.4.0.

An alignment of the *Pab* PolII intein and the *Pho* PolII intein dimer suggests low RMSD values between backbone atoms, but only when the bottom monomer of the *Pho* PolII intein in orange in Fig. 8 is aligned with the *Pab* PolII intein one lobe at a time. In Fig. 8a, residues 6–81 of the *Pho* PolII intein are aligned with the *Pab* PolII intein to a backbone RMSD of 0.93 Å. In Fig. 8b, residues 87–151 of the monomer in orange are aligned with the *Pab* PolII intein with an RMSD of 1.21 Å. However, the orientation of the *Pab* PolII intein with respect to the monomer segment is slightly altered between the two alignments. This suggests that each lobe of the monomer is generally folded as it would be in a typical HINT domain, but the two lobes do not combine properly to make an active unit.

Inteins have been described that fold as domain-swapped dimers and facilitate intein-mediated protein alternative splicing, or iPAS^28^. The naturally split *Nostoc punctiforme (Npu)* DnaE was converted into a fused *cis*-splicing intein, and an NMR structure was determined of a monomeric structure via NMR^29^. However, a variant with a three-residue deletion in the NCR and a one-residue deletion in the previous loop was found to form a domain swapped dimer (PDB 4KLS). However, in the case of the *Npu* DnaE structure, there is only one passage between N- and C-terminal domains, such that each HINT unit has its N-terminus from one monomer and its C-terminus from the other monomer. The three-dimensional swapping occurs within the NCR. Given that the active sites of each HINT unit come from a different monomer, the N- and C-exteins also come from a different monomer, allowing for alternative protein splicing. However, in the case of the *Pho* PolII structure, iPAS is very unlikely to occur, given that there are two passages between each domain such that the N- and C-termini of the HINT unit comes from the same monomer.

Regardless, we did test for alternative splicing with the *Pho* PolII intein. As described in the “Materials and Methods” section, we separately expressed the intein fusion protein with the truncated N-extein either in rich media or in minimal media supplemented with ^15^N-labeled ammonium chloride. The precursor proteins were mixed in a 1:1 ratio and allowed to splice. We analyzed the results via MALDI-TOF mass spectrometry (Fig. S6). We were able to identify peaks consistent with the masses of the ligated exteins from each monomer, but found no evidence of peaks for mixed products. This does not prove that iPAS does not occur, but it appears unlikely given both the orientation of the monomers and the structure of the active site. Instead, it seems more likely that there is an equilibrium in solution between a properly folded monomer and the domain swapped dimer, with a folded version of the *Pho* PolII intein with the typical HINT domain as the active species. We attempted to use dynamic light scattering and analytical ultracentrifugation to determine if there was evidence of a dimer in solution, but the protein concentrations required for those techniques resulted in high order aggregation of the protein. It is possible that the dimer we observe in the crystal structure is a function of crystal packing and not relevant in solution.

Our hypothesis was that the shorter NCR in the *Pho* PolII intein would promote splicing at a higher temperature than for the *Pab* PolII intein, due to increased rigidity of the intein fold. Our predictions in terms of activity appear to be correct, as splicing is less efficient at lower temperatures for the *Pho* PolII intein than for the *Pab* PolII intein, and the *Pho* PolII intein is less susceptible to cleavage by thermolysin. However, we have not been able to solve the structure of what we presume is the unspliced *Pho* PolII precursor, and it is possible that the higher temperature for efficient splicing could be required to unfolded any unproductive dimer in solution such that the intein can sample the proper unimolecular fold. One might speculate that a reservoir of inactive, misfolded dimer may exist in a biological context at suboptimal growth temperatures, and splicing could be conditionally triggered by an increase to the physiologically high temperature that then facilitates the unfolding of the trapped dimer and promotes the splicing needed to produce a functional DNA polymerase under optimal growth temperatures.

Our results do suggest that the proper folding of the intein can be influenced by the NCR region, and such observations are not unusual in the intein literature. For instance, the *Mycobacterium tuberculosis (Mtu)* RecA intein is interrupted by a HEN domain. This HEN domain has been deleted genetically to produce a *trans-*splicing intein, but *trans*-splicing requires denaturation and renaturation to generate a functional intein^30^. A *Mtu* RecA mini-intein that splices in *cis* were designed by replacing the HEN domain with an NCR^8^, but for splicing to proceed efficiently the intein required rounds of optimization by directed evolution^31^. Subsequent experiments suggested that the mutations selected in the directed evolution experiments were key to proper folding of the intein, in terms of both the necessary flexibility in the NCR and the stability of the fold overall to create a functional active site that properly coordinates the two symmetry-related units^21,31–35^. Likewise, deletion of the central region of the *Synechocystis* sp. PCC6803 (*Ssp*) DnaB intein resulted in an intein that could promote splicing^3^, but was improved in splicing efficiency by directed evolution experiments selecting for activating mutations throughout the intein^36^.

The *Pho* RadA intein is a native mini-intein with an NCR, and its structure was solved by both NMR and x-ray crystallography^37^. The NMR and crystal structures were highly similar except in the region of the NCR. The authors used this observation to create a minimized version of the intein, deleting 10 residues in the NCR. The intein splices efficiently with non-native exteins and the NCR region is well defined in the crystal structure of the minimized intein^37^. However, another group showed that the splicing activity of the NCR-minimized *Pho* RadA intein was compromised when inserted between the native exteins, and that the addition of ssDNA could rescue splicing activity^6^. This suggests that the length and flexibility of the NCR is important to the proper coordination of the active site, and that inteins interrupting non-native reporter exteins may not always reflect native splicing requirements. For example, the non-active dimer we observe may not occur if the intein were to interrupt the native *Pho* PolII protein, which might serve as an intramolecular chaperone to drive proper folding of the intein, which is inserted near the PolII active site.

In addition to iPAS facilitated by the variant of the *Npu* DnaE intein described above, iPAS also was observed by the incubation of a split C-terminal fragment of the *Methanocaldococcus jannaschii* (*Mja*) TFIIB intein with the native intein, by which the C-intein fragment inserts into the active site of the intact intein, displacing the native C-terminal segment of the intein^28^. The split site in the intein is adjacent to the NCR, suggesting that there is flexibility in this region of the intein that might promote dissociation between the N- and C-terminal segments of the folded native intein, as we observe in our structure. This led the authors to design NCR variants of the *Mja* TFIIB intein^11^. One inactive variant results in the insertion of an extra β-strand into the core of the intein, leading the authors to suggest that the interaction between the two symmetry-related intein units could be weak. The structure of the *Pho* CDC21-1 mini-intein suggests that residues in the NCR might enhance the interaction between the two symmetry-related units more so than in the *Pab* CDC21-1 intein, which has a shorter NCR^38^. We speculate that weak interaction between the units is also likely for the *Pho* PolII intein, that a native HEN domain or a longer NCR might serve to properly coordinate the two intein segments, and that native mini-inteins might be particularly susceptible to misfolding or alternative folding, particularly when expressed between non-native exteins.

## Materials and methods

### Plasmid preparation

Plasmid pPolWT was previously described and codes for the expression of a fusion protein of N-terminal *E. coli* maltose binding protein, the seven C-terminal residues of the *P. abyssi* PolII N-extein, the 185 residue intein, and the C-terminal sequence C-D-G-D-E-D-His_6_^4^. We rename that plasmid pMIHPab here for clarity. An additional XmnI site was introduced into pMIHPab with primers XmnU/XmnL via site-directed mutagenesis to make pMIHPabXmn. (The sequence of oligonucleotide primers are given in Supplementary Table S1.) Genomic DNA from *Pyrococcus horikoshii* strain JCM9974^39^ was obtained from ATCC. The gene for the *P. horikoshii* PolII intein was amplified by PCR with primers PhoPCRU and PhoPCRL, and was inserted between the XmaI and XmnI sites of pMIHPabXmn to make pMIHPhoXmn. Plasmid pMIHPho was created by digesting pMIHPhoXmn with XmnI and HindIII and inserting the annealed oligonucleotides PhoFU and PhoFL.

The resulting plasmid pMIHPho encodes for a fusion protein of N-terminal *E. coli* maltose binding protein, the seven C-terminal residues of the *P. horikoshii* PolII N-extein, the 166 residue intein, and the C-terminal sequence C-D-G-D-E-D-His_6_. In order to study splicing with a Gln166Asn mutation, we created pMIHPhoQN via site directed mutagenesis with oligonucleotides PhoQNU and PhoQNL. In order to study N-terminal cleavage uncoupled from splicing by changing residues Gln166 and Cys + 1 to Ala, we created pMIHPhoQACA via site directed mutagenesis with primers PhoQACAU and PhoQACAL. Corresponding mutants for pMIHPab have been previously described^4^.

To create a version of the wild-type *P. horikoshii* intein with a shorter N-extein, we created plasmid pNIHPho, which expresses an intein fusion protein with an N-extein of M-H-A-A-K-R-N fused to the 166-residue intein, and the C-extein C-D-G-D-E-D-His_6_. We generated pNIHPho from pMIHPho by making pMIHPhoNde via site directed mutagenesis with primers PhoNdeU and PhoNdeL to create a second NdeI site, digested with NdeI, and annealed the digested vector to make pNIHPho. pNIHQN and pNIHQACA were made in the same manner from pMIHQN and pMIHQACA, respectively. To insert a Cys1Ala mutation into pNIHPhoQACA and pNIHPhoQN, site directed mutagenesis was performed with oligonucleotide primers PhoNC1AU and PhoNC1AL. Similar plasmids for the *P. abyssi* intein have been previously described^15^.

### Protein expression and purification

To study the biochemical activity of intein-containing fusion proteins, appropriate plasmids were over-expressed in *E. coli* BL21(DE3) via induction at mid-log phase with 1 mM isopropyl-β-d-1-thiogalactopyranoside (IPTG) for 16 h at 20 °C with shaking. Protein pellets were lysed with buffer A (20 mM HEPES, pH 7.5, 500 mM NaCl) supplemented with 0.1 mM phenylmethylsulfonyl fluoride, 10 units of benzonase nuclease, an EDTA-free cocktail of protease inhibitors (Sigma-Aldrich), and 1 X BugBuster (Novagen), with incubation on ice for 30 min. Proteins with a His_6_-tag were purified using His-Link resin (Promega) with elution using buffer A supplemented with 250 mM imidazole. Proteins were concentrated and/or exchanged into buffer A using a centrifugal filter with a 3000 MWCO (Millipore), and final protein concentration was determined via a Bradford assay^40^.

In order to overexpress protein substituted with selenomethionine, we used B834(DE3) cells (Novagen), a methionine auxotroph, in place of BL21(DE3). Overnight cultures were prepared in rich LB media for 16 h at 37 °C, and a 2% inoculum was grown in LB media for 3 h. Cells were pelleted, and washed and resuspended in SelenoMet Medium Base (Molecular Dimensions), a supplemented M9 media including glucose but lacking methionine, and supplemented with SelenoMet nutrient mix (Molecular Dimensions). Cells were grown to mid log phase and induced with 1 mM IPTG and grown for 16 h at 20 °C.

To label NIHPhoQN for mass spectrometry analysis, we used M9 minimal media in place of LB media, and supplemented with 2 mM magnesium sulfate, 0.1 mM calcium chloride, 0.4% glucose, and 1 g/L ^15^N-labelled ammonium chloride.

### Intein activity assays

To study splicing, the MIH-QN version of the *P. abyssi* or *P. horikoshii* intein fusion protein was used with mutation of the C-terminal Gln to Asn to facilitate faster protein splicing^12^. Intein fusion protein at a concentration of about 0.2 µM was incubated in buffer A supplemented with 2 mM tris(2-carboxyethyl)phosphine (TCEP) and 5 mM EDTA for 8 h at the temperatures indicated in Fig. 2a. Splicing was analyzed by SDS-PAGE stained with Coomassie blue with four experiments per temperature using the same initial protein (Supplementary Fig. S1). The percentage of N-terminal cleavage was calculated using densitometry data from ImageJ, using the formula 100* MH/(MH + MIH). Splicing yields MH (45.2 kDa) and I (21.5 kDa for Pab, 19.2 kDa for Pho) from precursor MIH (66.7 kDa for Pab, 64.4 kDa for Pho).

To study N-terminal cleavage, the MIH-QACA version of the *P. abyssi* or *P. horikoshii* intein fusion protein was used to prevent splicing steps 2 (Cys + 1Ala) and 3 (C-terminal Gln to Ala). Intein fusion protein at a concentration of about 0.2 µM was incubated in buffer A supplemented with 100 mM dithiothreitol and 5 mM EDTA for 2 h at the temperatures indicated in Fig. 2b. N-terminal cleavage was analyzed by SDS-PAGE stained with Coomassie blue with four experiments per temperature using the same initial protein (Supplementary Fig. S2). The percentage of N-terminal cleavage was calculated using densitometry data from ImageJ, using the formula 100* M/(M + MIH). N-terminal cleavage yields M (43.7 kDa) and IH (23.0 kDa for Pab, 20.6 kDa for Pho) from precursor MIH (66.7 kDa for Pab, 64.3 kDa for Pho).

To study C-terminal cleavage, the NIH-C1AQN version of the *P. abyssi* or *P. horikoshii* intein fusion protein was used to prevent step 1 (Cys1Ala) and promote faster C-terminal cleavage with C-terminal Asn. A short N-extein was used such that it was easier to distinguish NIH from NI by SDS-PAGE. Intein fusion protein at a concentration of about 0.2 µM was incubated in buffer A supplemented with 2 mM TCEP and 5 mM EDTA for 5 h at the temperatures indicated in Fig. 2c. C-terminal was analyzed by SDS-PAGE stained with Coomassie blue with four experiments per temperature using the same initial protein (Supplementary Fig. S3). The percentage of C-terminal cleavage was calculated using densitometry data from ImageJ, using the formula 100* NI/(NI + NIH). C-terminal cleavage yields NI (22.4 kDa for Pab, 20.1 kDa for Pho) and H (1.5 kDa, too small for gel) from precursor NIH (23.9 kDa for Pab, 21.6 kDa for Pho).

### Intein structural stability assays

To study the relative strength of the disulfide bonds between Cys1 and Cys + 1 of the inteins, we used the precursor fusion proteins MIHPho and MIHPab. Purified unspliced precursor proteins were incubated in buffer A supplemented with the indicated concentrations of DTT for 30 min at 20 °C. The percentage of reduced protein was calculated using densitometry data from ImageJ, using the formula 100* MIH-red/(MIH-red + MIH-ox).

To study the susceptibility of the inteins to cleavage by the heat-stable protease thermolysin, we used the precursor fusion protein NIH-C1A-QACA of each intein, with mutations of Cys1, Cys + 1, and the C-terminal Gln to Ala to prevent all three steps of protein splicing, and the shorter N-extein to preclude digestion of the MBP. We incubated 2 µM protein in buffer C (50 mM Tris, pH 7.5, 2 mM CaCl_2_, 2 mM MgCl_2_, 5% glycerol), first for 10 min and then for 1 h after the addition of 30 ng/mL thermolysin at the indicated temperatures, and quenched the reaction with addition of EDTA to a final concentration of 30 mM.

To examine if the NIHPho-QN intein can promote alternative splicing, we purified unspliced precursor protein from over-expression in either rich LB media or M9 minimal media with N-15 labelled ammonium chloride. We purified the precursor proteins and exchanged them into Buffer A separately, mixed them in a 1:1 stoichiometric ratio, and incubated the mixture in buffer A supplemented with 2 mM TCEP and 5 mM EDTA for 16 h at 60 °C. The samples were analyzed by the multi-user mass spectrometry lab at CalTech and analyzed by MALDI-TOF mass spectrometry using α-Cyano-4-hydroxycinnamic acid as the matrix after desalting via a C4 Zip-tip.

### Structure analysis

The contact area of the dimer interface was calculated with the PyMOL function “get_area”. The contact area was calculated as the difference between the sum of the two monomer surface area, and the dimer surface area. Structural alignment was carried out using the PyMOL function “align,” while selecting relevant parts of the proteins.

### Crystallography conditions and structure determination

For crystallographic studies, protein NIHPho was used. Protein was purified as above, and exchanged into buffer X (20 mM Tris, pH 8.0, 100 mM NaCl). Final protein concentration was about 3 mg/ml. Crystallization conditions were screened using the sitting drop vapor diffusion method using a micro-bridge and Crystal Screen and Crystal Screen 2 (Hampton Research). The crystals were formed with a reservoir solution of 0.1 M Tris HCl, pH 8.5, with 1.8 M ammonium sulfate, 0.1 M sodium chloride, and 1% glycerol, with a mixture of 4 µl protein (about 3 mg/ml) and 1 µl reservoir solution.

Prior to data collection, all crystals were transferred to a cryo-protectant solution containing crystallization buffer with a glycerol concentration of 25%. The crystals were flash-cooled directly in liquid nitrogen. Diffraction data for the native and Se-Met derivative crystals were collected at 100 K using a MAR325 detector at the BL14-1 beamline of the Stanford Synchrotron Radiation Laboratory (SSRL). Data were processed, scaled, and reduced using the programs HKL2000^41^ and PHENIX suite^42^. The structure of the Se-Met *Pho* PolII intein was determined using diffraction data collect at the Se peak by single-wavelength anomalous diffraction method with the PHENIX suite, which automatically built about 60% of the *Pho* PolII intein structure. The native *Pho* PolII structure was refined based on the partial Se-Met derivative structure. The remaining 40% of the structure was manually fitted into the electron density map using Coot^43^. The completed structure was further iteratively refined using the PHENIX suite and manually adjusted and monitored with Coot (Supplementary Table S2). A sample of the fitting of the structure to the 2Fo-Fc electron density map at the domain swap junction region is given in Supplementary Fig. S7. The final Pho PolII intein structure was deposited with the Worldwide Protein Data Bank under PDB ID: 5BKH.

## Supplementary Information

Supplementary Information.

## Abbreviations

DTT: Dithiothreitol
EDTA: Ethylenediaminetetraacetic acid
HEN: Homing endonuclease
IPTG: Isopropyl-1-β-d-thiogalactopryanoside
MBP: *E. coli* Maltose binding protein
NCR: Non-conserved region (replaces HEN domain in mini-intein)
*Pab*: *Pyrococcus abyssi*
*Pho*: *Pyrococcus horikoshii*
TCEP: Tris(2-carboxyethyl)phosphine
