# Supplementary material for: An alternative domain-swapped structure of the Pyrococcus horikoshii PolII mini-intein
Source: Sci Rep. 2021 Jun 3;11:11680. doi: 10.1038/s41598-021-91090-w (PMC8175363; doi:10.1038/s41598-021-91090-w)
Supplement: Supplementary file 1 — Supplementary Information. [file 41598_2021_91090_MOESM1_ESM.pdf]

An alternative domain-swapped structure of the *Pyrococcus horikoshii* PolII mini-intein

Jennie E. Williams[1], Mario V. Jaramillo[1], Zhong Li[2,3], Jing Zhao[4], Chunyu Wang[4], Hongmin Li[2,3,5], and Kenneth V. Mills[1*]

1. Department of Chemistry, College of the Holy Cross, Worcester, MA, USA
2. Division of Genetics, Wadsworth Center, New York State Department of Health, Albany, NY 12208, USA.
3. Department of Pharmacology and Toxicology, College of Pharmacy, University of Arizona, Tucson, AZ 85721, USA.
4. Center for Biotechnology and Interdisciplinary Studies, Rensselaer Polytechnic Institute, Troy, NY, USA.
5. Department of Biomedical Sciences, School of Public Health, University at Albany, Albany, NY, USA.

Supplementary Figure S1. Splicing of PolII inteins from *Pyrococcus horikoshii* and *Pyrococcus abyssi*.

Supplementary Figure S2. N-terminal cleavage of PolII inteins from *Pyrococcus horikoshii* and *Pyrococcus abyssi*.

Supplementary Figure S3. C-terminal cleavage activity of PolII inteins from *Pyrococcus horikoshii* and *Pyrococcus abyssi*.

Supplementary Figure S4. Thermolysin analysis of PolII inteins from Pyrococcus horikoshii and Pyrococcus abyssi.

Supplementary Figure S5. Disulfide bond strength of PolII inteins from Pyrococcus horikoshii and Pyrococcus abyssi.

Supplementary Figure S6. MALDI-TOF MS analysis of protein splicing.

Supplementary Figure S7. The 2Fo-Fc electron density map at the domain swap junction region.

Supplementary Table S1. Oligonucleotides used in plasmid construction.

Supplementary Table S2. Data collection, phasing and refinement statistics

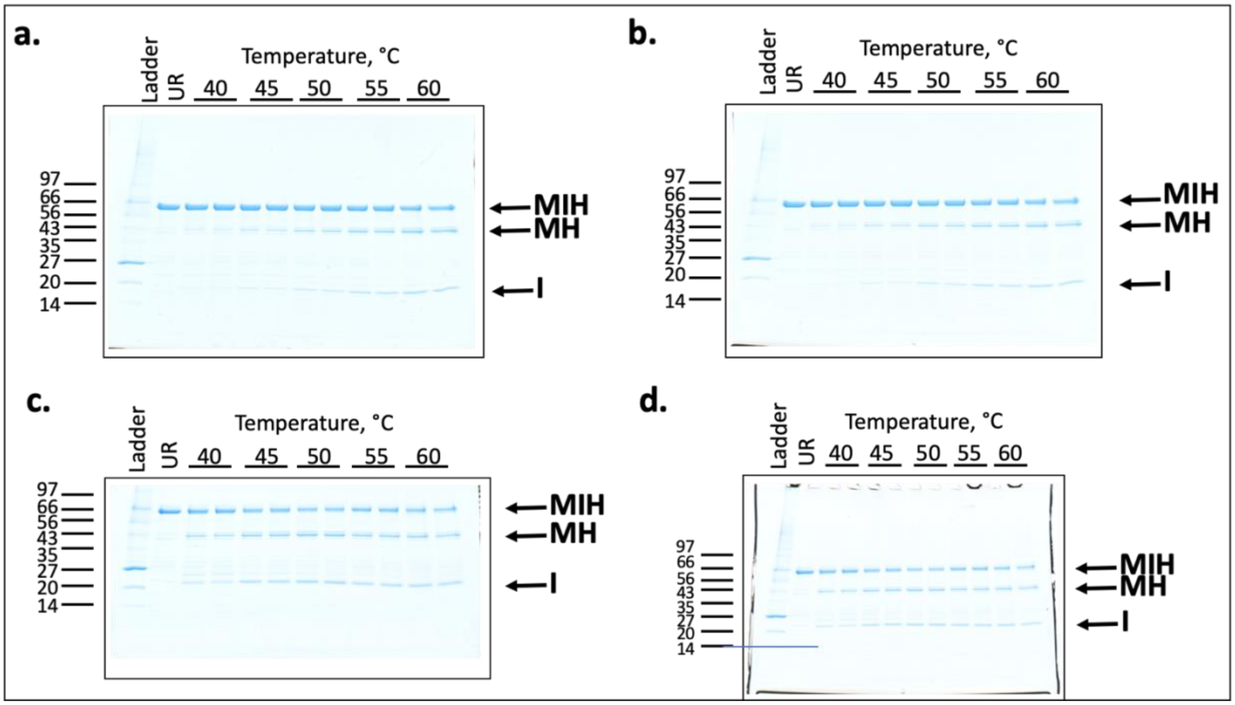

Supplementary Figure S1. Splicing of PolII inteins from *Pyrococcus horikoshii* and *Pyrococcus abyssi*.

SDS-PAGE analysis of protein splicing of intein fusion protein MIH-QN for *Pho* PolII (**a. and b.**) and *Pab* PolII (**c. and d.**). Protein splicing converts precursor MIH into spliced product MH and excised intein I. Four reactions were run at each temperature point from one purified precursor protein. The extent of each reaction was measured by SDS-PAGE stained with Coomassie blue, with analysis of percentage of activity by densitometry using ImageJ. Precursor protein MIH-QN was incubated in buffer A supplemented with 2 mM TCEP and 5 mM EDTA for 8 h at the indicated temperatures. UR is unreacted precursor protein.

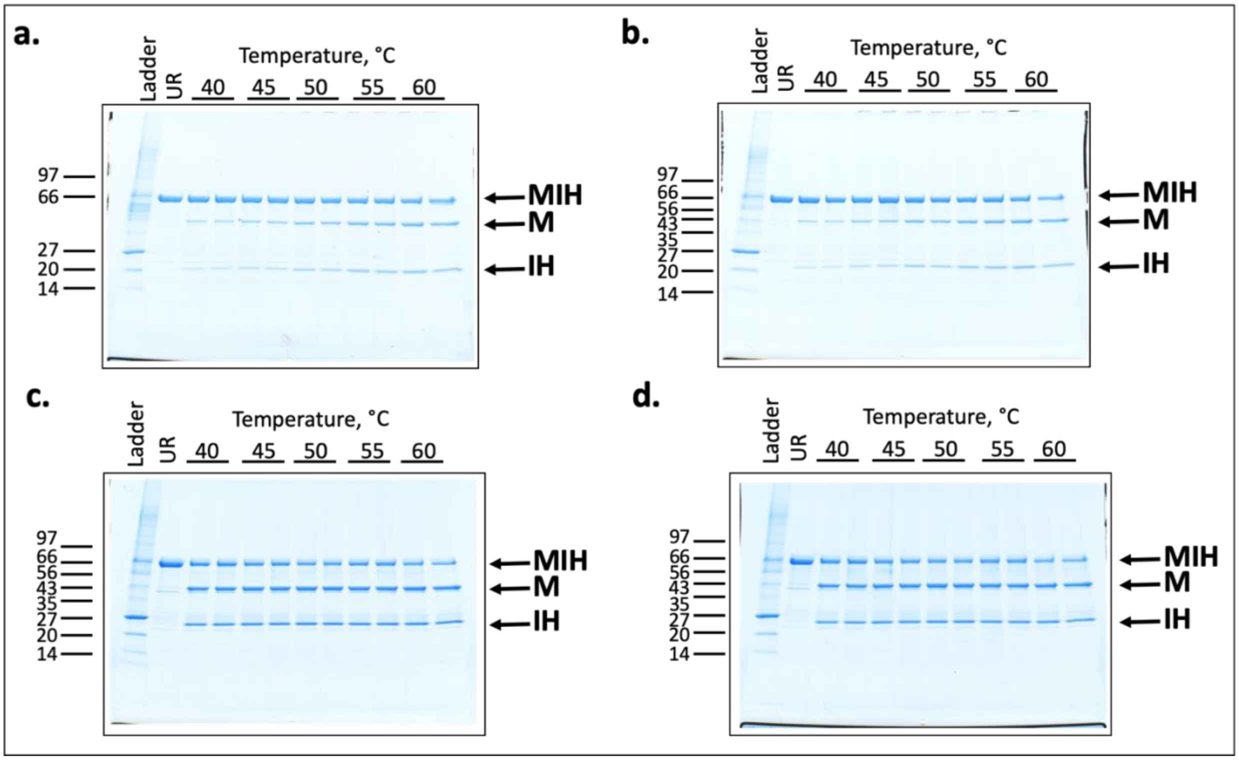

Supplementary Figure S2. N-terminal cleavage of PolII inteins from *Pyrococcus horikoshii* and *Pyrococcus abyssi*.

SDS-PAGE analysis of N-terminal cleavage of intein fusion protein MIH-QACA for *Pho* PolII (**a. and b.**) and *Pab* PolII (**c. and d.**). N-terminal cleavage converts precursor protein MIH into M and IH. Four reactions were run at each temperature point from one purified precursor protein. The extent of each reaction was measured by SDS-PAGE stained with Coomassie blue, with analysis of percentage of activity by densitometry using ImageJ. Precursor protein MIH-QACA was incubated in buffer A supplemented with 100 mM DTT and 5 mM EDTA for 2 h at the indicated temperatures. UR is unreacted precursor protein.

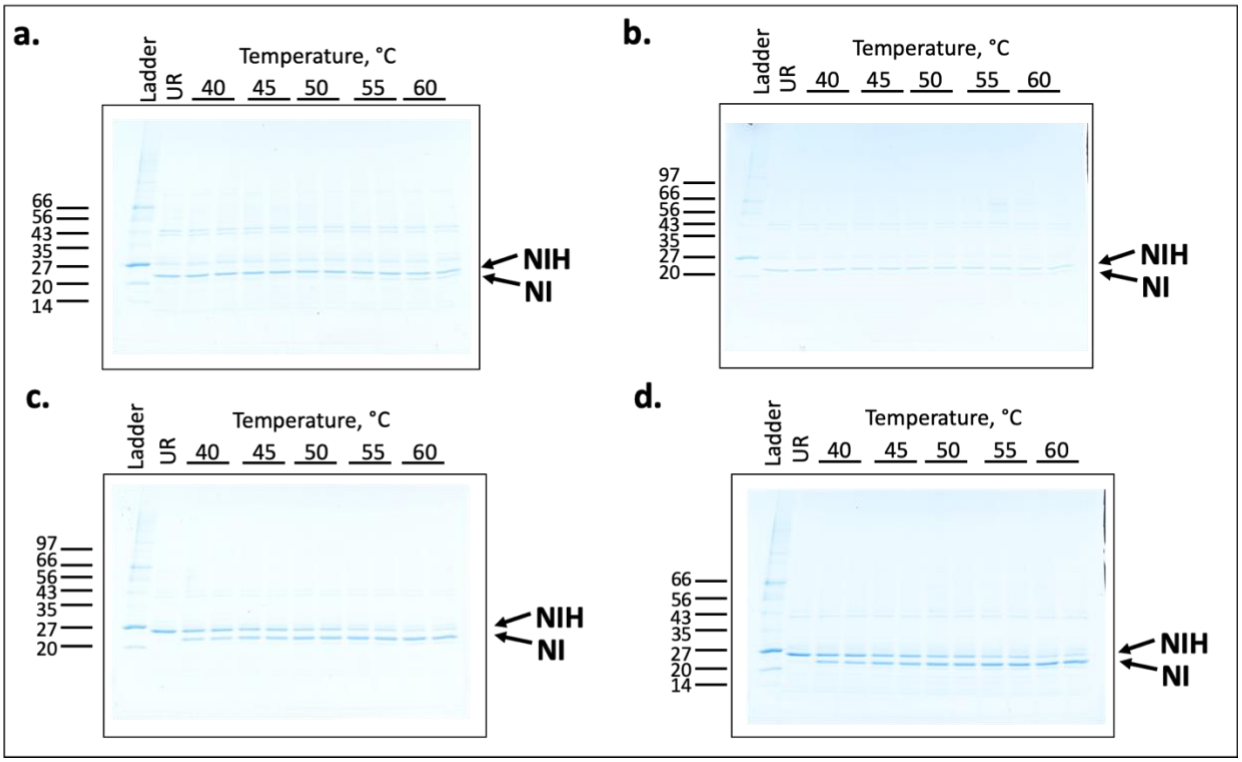

Supplementary Figure S3. C-terminal cleavage activity of PolII inteins from *Pyrococcus horikoshii* and *Pyrococcus abyssi*.

SDS-PAGE analysis of C-terminal cleavage of intein fusion protein NIH-C1A-QN for *Pho* PolII (**a. and b.**) and *Pab* PolII (**c. and d.**). C-terminal cleavage converts precursor protein NIH into NI and H, the latter of which is too small to observe on the gel. Four reactions were run at each temperature point from one purified precursor protein. The extent of each reaction was measured by SDS-PAGE stained with Coomassie blue, with analysis of percentage of activity by densitometry using ImageJ. Precursor protein NIH-C1A-QN was incubated in buffer A supplemented with 2 mM TCEP and 5 mM EDTA for 5 h at the indicated temperatures. UR is unreacted precursor protein.

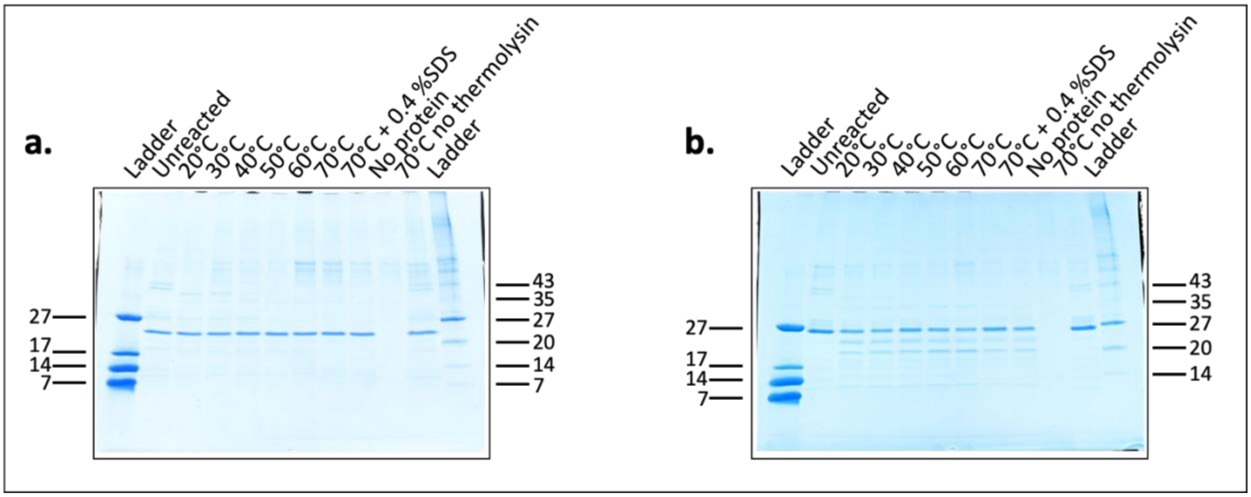

Supplementary Figure S4. Thermolysin analysis of PolII inteins from *Pyrococcus horikoshii* and *Pyrococcus abyssi*.

SDS-PAGE analysis, stained with Coomassie blue, of the susceptibility of the *Pho* PolII intein (**a.**) and *Pab* PolII intein (**b.**) to cleavage by the heat-stable protease thermolysin. We used the precursor fusion protein NIH-C1A-QACA of each intein, with mutations of Cys1, Cys+1, and the C-terminal Gln to Ala to prevent all three steps of protein splicing, and the shorter N-extein to preclude digestion of the MBP. We incubated 2 µM protein in buffer C (50 mM Tris, pH 7.5, 2 mM $CaCl_2$, 2 mM $MgCl_2$, 5% glycerol), first for 10 minutes and then for 1 hour after the addition of 30 ng/mL thermolysin at the indicated temperatures, and quenched the reaction with addition of EDTA to a final concentration of 30 mM.

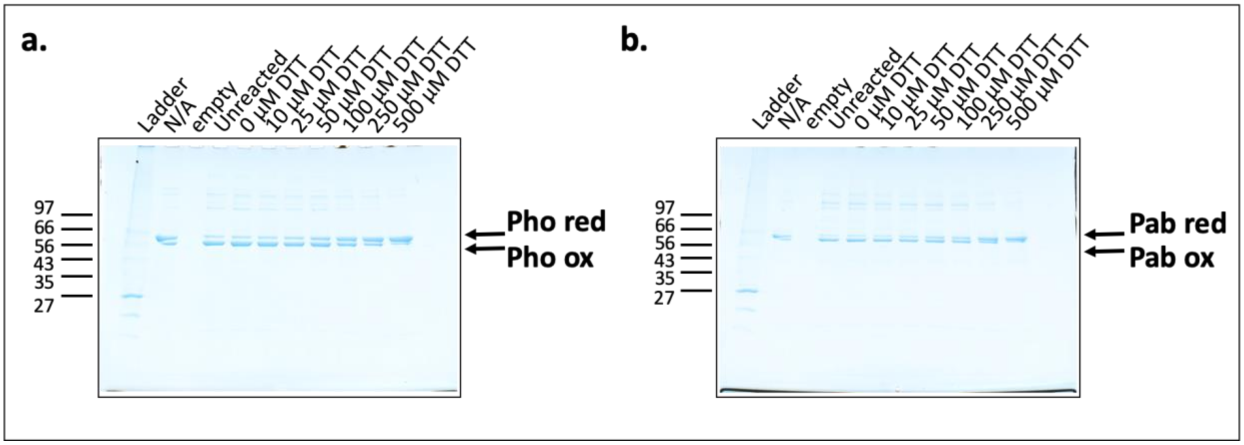

Supplementary Figure S5. Disulfide bond strength of PolII inteins from *Pyrococcus horikoshii* and *Pyrococcus abyssi.*

SDS-PAGE analysis, stained with Coomassie blue, of the reduction of the disulfide bond between Cys1 and Cys+1 of the *Pho* PolII intein (**a.**) and *Pab* PolII intein (**b.**). To study the relative strength of the disulfide bonds, we used MIHPho and MIHPab. Purified unspliced precursor proteins were incubated in buffer A supplemented with the indicated concentrations of DTT for 30 min at 20°C. The percentage of reduced protein was calculated using densitometry data from ImageJ, using the formula 100* MIH-red/(MIH-red + MIH-ox).

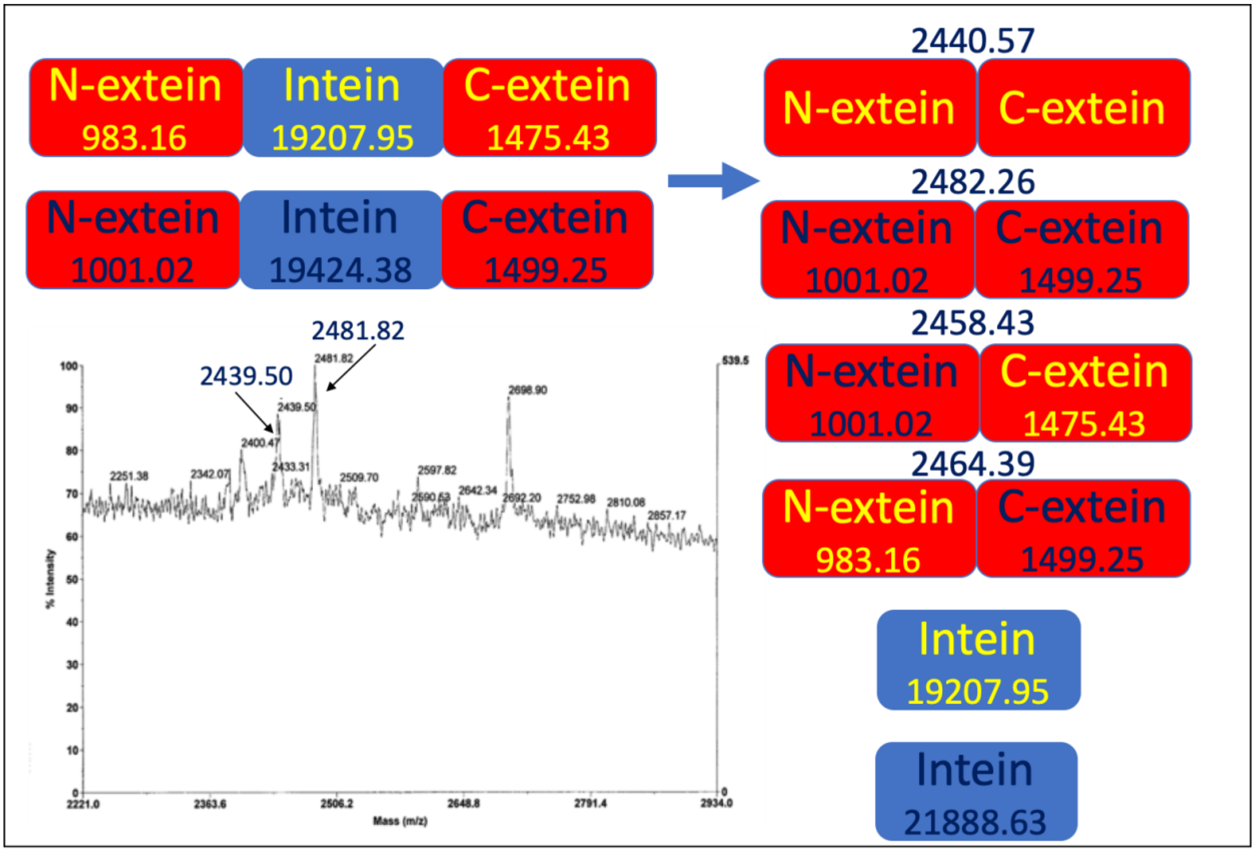

Supplementary Figure S6. MALDI-TOF MS analysis of protein splicing. Protein MIHPho-QN was grown in both rich media and in M9 media supplemented with N-15 labelled ammonium chloride. Labelled and unlabeled protein were purified and exchanged into buffer A as described in the *Materials and Methods* and incubated in a 1:1 stoichiometric ratio for 16 h at 60°C in buffer A supplemented with 2 mM TCEP and 5 mM EDTA. The resulting mixture was desalted via ZipTip and analyzed by MALDI-TOF MS. The predicted masses are given in the cartoon in the figure, with the red labels indicating Mr for unlabeled proteins and the black labels indicating Mr for the labeled proteins, with the Mr for the four possible spliced products above the cartoons.

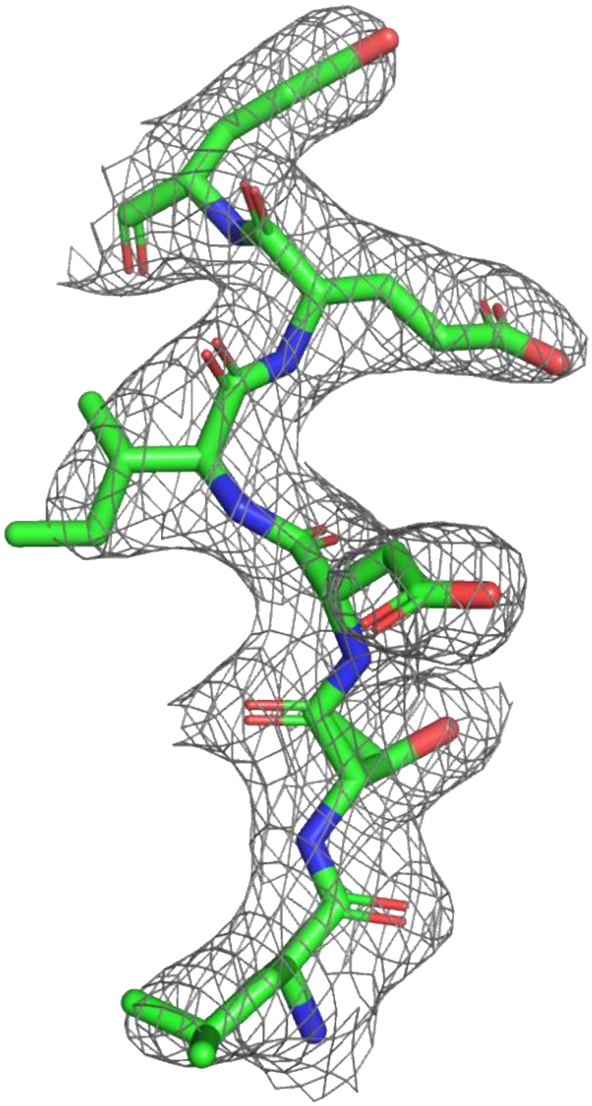

Supplementary Figure S7. The 2Fo-Fc electron density map at the domain swap junction region. Map was contoured at 1.0δ level.

Supplementary Table S1. Oligonucleotides used in plasmid construction.

XmnU, 5’-CGTTATAATAAATGAAAATATTCTAACGCATCAATGTGATGGCG
XmnL, 5’-CGCCATCACATTGATGCGTTAGAATATTTTCATTTATTATAACG

PhoPCRU, 5’-CAAAGAGGAGAAATTGCTTCCCGGGAGAT
PhoPCRL, 5’-TCGCACTGATGCGTCAGAATATTTTCATTA

PhoFU, 5’-
TATTGTAACGCATCAATGTGATGGCGATGAAGACCACCACCACCACCACTGA
PhoFL, 5’-
AGCTTCAGTGGTGGTGGTGGTGGTCTTCATCGCCATCACATTGATGCGTTACAA
TA

PhoQNU, 5’-
GTAATAATTAATGAAAATATTGTAACGCATAATTGTGATGGCGATGAAGACCACC
PhoQNL, 5’-
GGTGGTCTTCATCGCCATCACAATTATGCGTTACAATATTTTCATTAATTATTAC

PhoQACAU, 5’-
CTGTAATAATTAATGAAAATATTGTAACGCATGCAGCTGATGGCGATGAAGACCAC
CA
PhoQACAL, 5’-
TGGTGGTCTTCATCGCCATCAGCTGCATGCGTTACAATATTTTCATTAATTATTACAG

PhoNC1AU, 5’-GCTGCGAAGAGGAGAAACGCCTTCCCGGGAGA
PhoNC1AL, 5’-TCTCCCGGGAAGGCGTTTCTCCTCTTCGCAGC

PhoNdeU, 5’-GGATCGAGGGAAGGCCTCATATGCATGCTGCGAAGAG
PhoNdeL, 5’-CTCTTCGCAGCATGCATATGAGGCCTTCCCTCGATCC

Supplementary Table S2. Data collection, phasing and refinement statistics.

	Nat[a]	Se-Met[a]
Data Collection		
Wavelength (Å)	0.97907	0.97907
Space group	*P* 6₃ 2 2	*P* 6₃ 2 2
Unit cell		
a, b, c (Å)	128.93, 128.93, 79.25	128.62, 128.62, 79.40
α, β, γ (°)	90 90 120	90 90 120
Resolution range	37.25-2.43 (2.52-2.43)[b]	45.59-2.64 (2.74-2.64)
Total reflections	183,962 (17666)	210,806 (20891)
Unique reflections	13,650 (1318)	11,815 (1149)
Multiplicity	13.5 (13.4)	17.8 (18.2)
Completeness (%)	90.17 (89.54)	99.86 (99.22)
Mean I/sigma(I)	32.75 (3.60)	37.71 (3.92)
Wilson B-factor	57.27	62.90
R-merge	0.07345 (0.88)	0.07396 (0.95)
R-meas	0.07626 (0.91)	0.07614 (0.98)
R-pim	0.0201 (0.24)	0.01789 (0.23)
CC1/2	0.999 (0.88)	1 (0.89)
CC*	1 (0.967)	1 (0.97)
R_{ano}		0.072
Se sites		5
Figure of Merit		0.21
Refinement		
Reflections		
For Refinement	13,646 (1,318)	
For R-free	1,365 (133)	
R_{work}	0.24 (0.40)	
R_{free}	0.27 (0.43)	
CC(work)	0.95 (0.73)	
CC(free)	0.94 (0.56)	
No. atoms		
Protein	1394	
Water	24	
Average B-factor (Å^2)	65.21	
macromolecules	65.28	
solvent	61.67	
RMS (bonds) (Å)	0.004	
RMS (angles) (°)	0.72	
Ramachandran		
favored (%)	95.83	
allowed (%)	4.17	
outliers (%)	0.00	

a. One crystal was used for each structure.
b. Statistics for the highest-resolution shell are shown in parentheses.
